# Human Gut-Associated Natural Killer Cells in Health and Disease

**DOI:** 10.3389/fimmu.2019.00961

**Published:** 2019-05-03

**Authors:** Alessandro Poggi, Roberto Benelli, Roberta Venè, Delfina Costa, Nicoletta Ferrari, Francesca Tosetti, Maria Raffaella Zocchi

**Affiliations:** ^1^Molecular Oncology and Angiogenesis Unit, IRCCS Ospedale Policlinico San Martino, Genoa, Italy; ^2^Immunology Unit, IRCCS Ospedale Policlinico San Martino, Genoa, Italy; ^3^Division of Immunology, Transplantation and Infectious Diseases, IRCCS San Raffaele Scientific Institute, Milan, Italy

**Keywords:** gut-associated lymphoid tissues, natural killer cells, innate lymphoid cells, inflammatory bowel disease, colorectal carcinoma

## Abstract

It is well established that natural killer (NK) cells are involved in both innate and adaptive immunity. Indeed, they can recognize molecules induced at the cell surface by stress signals and virus infections. The functions of NK cells in the gut are much more complex. Gut NK cells are not precisely organized in lymphoid aggregates but rather scattered in the epithelium or in the stroma, where they come in contact with a multitude of antigens derived from commensal or pathogenic microorganisms in addition to components of microbiota. Furthermore, NK cells in the bowel interact with several cell types, including epithelial cells, fibroblasts, macrophages, dendritic cells, and T lymphocytes, and contribute to the maintenance of immune homeostasis and development of efficient immune responses. NK cells have a key role in the response to intestinal bacterial infections, primarily through production of IFNγ, which can stimulate recruitment of additional NK cells from peripheral blood leading to amplification of the anti-bacterial immune response. Additionally, NK cells can have a role in the pathogenesis of gut autoimmune inflammatory bowel diseases (IBDs), such as Crohn's Disease and Ulcerative Colitis. These diseases are considered relevant to the generation of gastrointestinal malignancies. Indeed, the role of gut-associated NK cells in the immune response to bowel cancers is known. Thus, in the gut immune system, NK cells play a dual role, participating in both physiological and pathogenic processes. In this review, we will analyze the known functions of NK cells in the gut mucosa both in health and disease, focusing on the cross-talk among bowel microenvironment, epithelial barrier integrity, microbiota, and NK cells.

## Introduction

Gut associated lymphoid tissue (GALT), the part of the mucosa-associated lymphoid tissue (MALT) found along the gastrointestinal tract (GI), is essential to understanding the reaction of the host to external environment components (1–3). The content of gut lumen is continuously shucked and changed from the birth through adulthood and into old age. These changes strongly influence the type of immune response elicited and, consequently, may generate gut diseases (4–6). The GALT should be able to distinguish pathogenic and harmful organisms and antigens from those that are beneficial and not dangerous. The innate arm of the immune system can be considered the first line of defense against pathogenic microorganisms and their products; this arm can distinguish among microorganisms via pattern recognition receptors (PRRs) and Toll-like receptors (TLRs) present on macrophages, neutrophils, and dendritic cells (7–9). Also, natural killer (NK) cells can interact with gut microorganisms and influence and shape the adaptive T cell-mediated immune response by acting on professional antigen presenting cells (APCs), such as dendritic cells (DCs). The phenotypic and functional features of gut associated NK cells are different from those that are typical of NK cells isolated from peripheral blood. In addition, gut lymphoid cells expressing some markers of NK cells, termed innate lymphoid cells (ILC) (10–12), appear to be involved in the regulation of gut mucosa homeostasis and in the generation of gut-associated lymphoid structures (13–19). On this basis, it is conceivable that NK cells (ILC1) and other subsets of ILC (ILC2 and ILC3) are not only involved in the control of healthy gut but also in the pathogenesis and evolution of gut diseases, including inflammatory bowel disease (IBD) and colorectal carcinoma (CRC) (20–23).

## NK Cell Phenotypic and Functional Features in Healthy Gut Mucosa

It is well-established that NK cells represent a group of CD3^−^CD56^+^ innate immune cells (24). The majority of the findings on this topic is derived from studies on NK cells isolated from peripheral blood mononuclear cells (PBMCs) (24). Several comprehensive review articles have described the phenotypic and functional features of PB NK cells in detail (25–29). Briefly, PB NK cells can express the CD56 antigen at different intensities; indeed, CD56^bright^ and CD56^dull^ NK cells with predominant cytokine production or cytotoxic functions, respectively, have been identified. Several reports have stated that these two cell populations show a different functional role and a distinct array of receptors involved in the recognition of self-class I human histocompatibility antigens (HLA-I) (30–33). It is not clear whether these two NK cell subsets derive from the same or different NK cell precursors and whether they display some plasticity, converting from one into the other (34–36). Generally, lymphoid cells expressing CD56 isolated from the gut do not express CD16, i.e., the classical receptor for the crystallizable fragment of immunoglobulin (FcγRIIIa) that is usually found on majority of PB NK cells (24). It has been reported that CD3^−^CD56^+^ cells, like several other populations of tissue-resident innate cells (37–39), do not display a strong cytolytic activity if tested *in vitro* against conventional NK cell targets, but rather produce and release IFNγ *in vitro*. According to studies of human and murine NK cells, the reduction or lack of cytotoxicity can be considered as a marker of immaturity in the main function displayed by NK cells (40–42). More correctly, CD3^−^CD56^+^CD16^−^ mucosal NK cells could be considered lymphoid effectors that have a relevant role in the regulation of gut homeostasis (11). In line with this interpretation, the production of IL22 by GALT NK cells is essential for modulating expression of many genes in mucosal epithelial cells to favor epithelial cell survival and remodeling (40, 43). Of note, IL22 is produced by mucosal T lymphocytes, and it is conceivable that the imbalance between IL22 and IL17 is relevant to the generation of IBD (44). Importantly, GALT NK cells produce IL22 in response to IL23, but not to IL12. The more conventional NK cells found in PB or in the gut mucosa respond to IL12 by producing IFNγ (45); these cytokines are involved in the response to infections (42–46). NK cells were found in lamina propria (LP) scattered in the middle of epithelial cells as intraepithelial lymphocytes (IELs), but not associated with lymphoid aggregates. This suggests that these cells do not participate as actively as T cells at antigen inductive sites present in the gut (41, 47).

## Small and Large Intestine Mucosa Histology and Function

**Figure 1:**
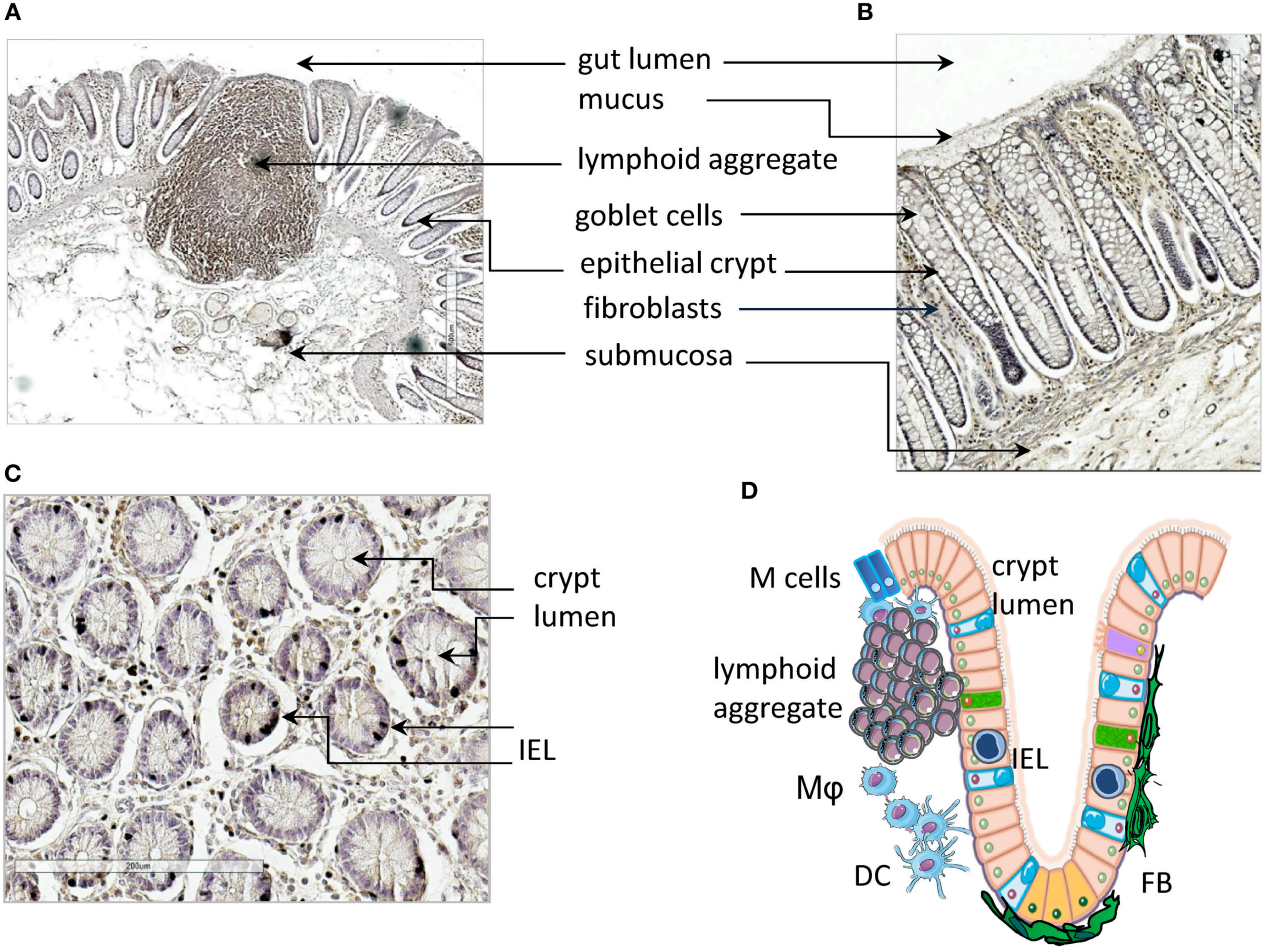
Gut mucosa structure and gut-associated lymphoid tissue (GALT). **(A–C)** Longitudinal **(A,B)** and transversal (s) sections of healthy gut showing a lymphoid aggregate **(A)**, identified by immunostaining with an anti-CD45RO monoclonal antibody, epithelial crypts **(A–C)**, fibroblasts **(B)** and intraepithelial lymphocytes (IELs, **C**). **(D)** Schematic representation of the structure of gut mucosa and GALT. IELs, intraepithelial lymphocytes; Mϕ, macrophages; DC, dendritic cells; FBs, fibroblasts. Arrows in **(A–C)** indicate the microscopic anatomy of gut mucosa, with the indicated cells and structures. Pink: enterocytes; yellow-orange: LGR5^+^ stem cells; cyan, goblet cells; purple, tuft cells; green, neuroendocrine cells; blue, M cells; IELs, intraepithelial lymphocytes.

Herein, we focus mainly on the mucosa of the small intestine where the first interaction with lumen content takes place (Figures 1A,B). The surface epithelium and the underlying LP are arranged in villi and crypts, which give a velvet-like appearance to the mucosa. These structures amplify the absorbent surface of the gut and the crypts are surrounded and reinforced by a strong sheath of fibroblasts (FBs) (Figure 1B). Lymphoid and myeloid cells present within the gut mucosa can interact with epithelial absorptive cells (enterocytes), goblet cells that secrete ions, water and mucus, and a few endocrine cells that produce hormones and neuropeptides (47, 48). Goblet cells and enterocytes are derived from undifferentiated cells in a close cross-talk with pericryptal FBs and this relationship enhances the structural integrity and functional efficiency of the gut mucosa (49, 50). Importantly, LP penetrates the villi cores associated with blood vessels, connective tissue with different myeloid cells, smooth muscle cells, and blind-ended lacteals (Figures 1A–C, scheme in Figure 1D). The mucosa of large intestine is composed of the same cell types, but the structural organization is different; indeed, it is quite smooth without villi, and goblet cells are outnumbered by columnar absorptive cells. The principal protection for excluding undesired environmental factors, particularly harmful microorganisms, is provided by the GALT. GALT is composed of lymphoid aggregates (Figure 1A), mucosal LP lymphocytes, and intraepithelial lymphoid cells (Figure 1C) (51–53). Lymphoid aggregates increase in number along the small intestine and become confluent in the ileum, giving rise to Peyer's patches. These are un-encapsulated lymphoid structures, similar to lymph nodes (LN), with follicles composed of different cell types, including B, T, and accessory myeloid cells such as DCs (53, 54). Peyer's patches are important sites for the induction of the immune response, and the overlying epithelium contains multi-fenestrated or microfold cells (M cells). M cells take up different molecules from the gut lumen that, through transcytosis, come in contact with the underlying lymphoid cells (55). In the LP there are plasma cells, which mainly produce IgA, that protect from bacterial invasion, macrophages, T and B lymphocytes, and polymorphs. Intraepithelial lymphocytes are concentrated in the small intestine, reaching a maximal ratio of 20 lymphocytes to 100 enterocytes in the jejunum and a lower ratio in the ileum (56). While the small intestine, less colonized by bacteria, shows both isolated lymphoid follicles (ILFs) and specialized Peyer's patches, only ILFs are observed in the colon. These ILFs are in variable positions but arise whenever bacterial products permeate the epithelial barrier. The assembly of ILFs has only been extensively elucidated in mouse models, where it is linked to lymphoid tissue inducer (LTi) cells. These cells are a subpopulation of ILC3 and depend on retinoic acid receptor-related orphan receptor gamma (RORγt) expression. It is likely that colorectal ILFs in humans can also arise *de novo*, as their density and grade of maturation vary considerably in any given area of mucosa (57).

## NK Cell Localization in the Gut

Although NK cells are present in the gut, it remains to be defined if these cells are resident or derive from PB circulating NK cells. It is well established that leukocytes can extravasate in a specific area under the chemotactic stimuli and action of adhesion molecules (58–61). For example, the lymphocyte function associated antigen 1 (LFA1, CD11a/CD18) can interact with the intercellular adhesion molecule (ICAM)-1 expressed on endothelial cells, while the CD29/CD49d integrin (formerly very late antigen-4) binds to the vascular cell adhesion molecule (VCAM)-1 (60, 61). Of note, ICAM1 and VCAM1 are usually absent in healthy endothelial cells; however, the effect of inflammatory cytokines, such as IL1β, IFNγ, and TNFα, in tissues where an immune response takes place, results in strong upregulation of both ICAM1 and VCAM1 (60–62). PB NK cells can express several chemokine receptors (63), some of which are more abundant on CD56^bright^ than on CD56^dull^ peripheral NK cells (64). It has been shown that *ex vivo* isolated NK cells bear CXCR1, CXCR3, and CXCR4, and contain subsets expressing CCR1, CCR4, CCR5, CCR6, CCR7, CCR9, CXCR5, and CXCR6. More precisely, CD56^dull^ NK cells display a repertoire of chemokine receptors similar to that of neutrophils while this repertoire in CD56^bright^ is most similar to that of T-helper (Th) 1 cells. These findings suggest that the CD56^dull^ and the CD56^bright^ PBNK cells can migrate into tissues either at the beginning of the inflammatory reaction, which accompanies the immune response, or later (65). Of note, both CD56^dull^ and CD56^bright^ PB NK cells do not express the chemokine receptors needed to home to the small intestine, such as CCR6 and CCR9 (64–66). The lack of this homing capability would suggest that NK cells found in the gut are not derived from PB NK cells. However, some PB NK cells can express the CD161 antigen, also called NKRP1A (67, 68). This receptor is upregulated on NK cells upon stimulation with IL2 and, more importantly, it is expressed on majority of intestinal infiltrating lymphocytes (68, 69), including NK cells and some subsets of ILC (2, 5, 10). It has been demonstrated that CD161 can function as an adhesion molecule involved in the transmigration of PB CD4^+^ T cells through endothelial cells (70). It is still unknown whether CD161 also plays a role in the transendothelial migration of PB NK cells, but it can be speculated that CD161^+^ PB NK cells localize in the tissue upon the cooperative involvement of LFA1, and engagement of the platelet endothelial cell adhesion molecule-1 (PECAM1/CD31) on NK cells. Indeed, most NK cells express CD31, which allows a homophilic interaction with the CD31 present at the endothelial junction (71–74). CD161 might also regulate the speed of migration, as was shown for CD4^+^CD161^+^ T lymphocytes (70). The stromal derived factor 1 (SDF1, also named CXCL12), recognized by CXCR4, appears to favor tissue localization of NK cells, in particular of the CD56^bright^ subset. However, NK cells, considered to be NKp46^+^ lymphocytes, are not so represented in the gut, although several chemokines are detectable in bowel diseases, including CRC (75, 76). Collectively, these findings indicate that PB NK cells may localize into the gut, but their origin and the relative contribution of adhesion molecules and chemokine receptor-ligand interactions are yet to be established. Table 1 summarizes the main surface molecules, and their respective ligands, involved in gut NK cell function.

**Table 1:** Main surface molecules involved in NK cell function in the gut.

**Molecule**	**Family**	**Function**	**Ligands**	**Disease**
CD56/NCAM	Immunoglobulins	Cell-cell, cell-matrix adhesion	HSPGs^a^, L1, NCAM	CD, CRC
CD57/GA3S^b^	Carbohydrates	Adhesion, NK cell maturation	nd	CRC
NKRP1A/CD161	C-type lectin	Cell-cell, cell-matrix adhesion	LLT1^c^	CRC
NKG2D/CD314	C-type lectin	Recognition of infected/ transformed cells	MICs^d^, ULBPs^e^	CD, UC, CRC
NKp30/CD337	NCR^f^	NK cell cytolytic activity	BAG6^g^, B7-H6, Gal-3	CRC
NKp44/CD336	NCR	NK cell cytolytic activity	HSPGs, MLL5^h^	CD
NKp46/CD335	NCR	NK cell polarization	HSPGs, HAN^i^	CD, CRC
KIRs/CD158	Immunoglobulin like	Regulation of NK cell activity	HLA-A,-B, -C, -G	CD, CRC

a *Heparansulfate proteoglycans*;

b *Glucuronic acid-3 sulfate*;

c *Lectin-like transcript-1*;

d *MHC-related molecules*;

e *UL16-binding proteins*;

f *Natural Cytotoxicity Receptors*;

g *BCL-2-associated athanogene-6*;

h *Mixed-lineage leukemia-5*;

i *Hemagglutinin-A neuraminidase*.

## Intestinal Crypt Niche and Immune System Cross-Talk as the First Line of defense in the Gut

**Figure 2:**
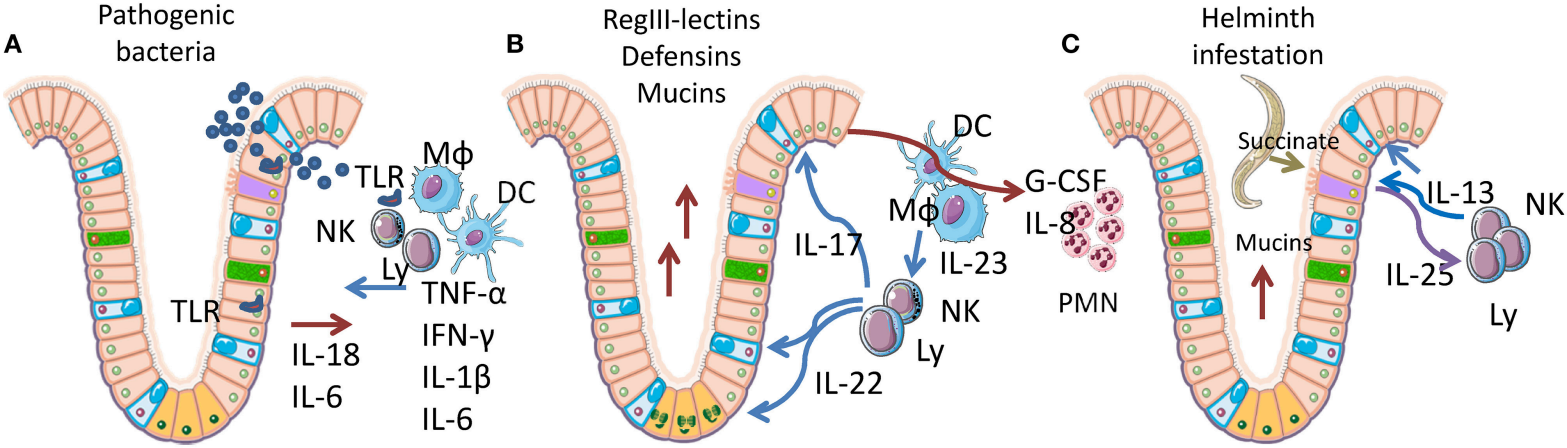
Epithelial-immune cell cross-talk in gut mucosa. Simplified representation of the cytokine networks activated by the response to bacteria or parasites. Pink, enterocytes; yellow-orange, LGR5^+^ stem cells; cyan, goblet cells; purple, tuft cells; green, neuroendocrine cells; Mϕ, macrophages; DC, dendritic cells; Ly, lymphocytes; NK, natural killer cells. **(A)** Acute inflammation caused by pathogenic bacteria is recognized through Toll-like receptors (TLR) and induces IL18 and IL6 release by IECs, followed by TNFα, IL1β, IFNγ, and IL6 release by lymphoid and myeloid cells. These cytokines trigger mucosal permeabilization and increase IEC proliferation and death. **(B)** Homeostatic inflammation, sustained by IL22 and IL17, secreted by lymphoid cells upon IL23 stimulation by myeloid cells. IL22 specifically targets IECs, restoring the epithelial barrier (tight junction formation), inducing the proliferation of LGR5^+^ stem cells in the crypt bottom. IL22 also induces hyperplasia in goblet cells, with increased mucins production. IL17 induces G-CSF and IL8 expression in IECs, fibroblasts and leucocytes causing neutrophils recruitment. **(C)** Parasite infection sensed by tuft cells, which are able to recognize succinate that is released by many helminths. Tuft cells react by expressing IL25, which induces IL13 release and activation of ILC2 cells. IL13 mediates proliferation of tuft cells (amplifying the response) and goblet cell activation, finally determining a Th2 response.

Intestinal crypts have the highest rate of tissue turnover in the body (3–5 days), a process that is strongly influenced by the products (mainly short-chain fatty acids and lactate) of commensal bacteria, (77, 78). As the crypt contains the proliferating component of mucosa (3–5 stem cells in each crypt), providing for self-renewal of the entire tissue, its niche is organized to limit mechanical, infectious, and inflammatory damage (Figure 2). LGR5-positive stem cells are positioned at the bottom of the crypt, mixed with Paneth cells in the small intestine. Paneth cells secrete both trophic (wnt3) and anti-microbial factors (alpha-defensins, lysozyme, RegIIIγ lectin) (Figure 2B). Goblet cells, mixed with maturating enterocytes inside the crypt, produce mucus and release antimicrobial molecules, thereby creating the main barrier to microbial infection. Under normal conditions, the inner mucus layer is almost microbe-free, thus neither epithelial nor immune cells are in contact with danger signals (79–81). TLRs act as the main sensors for pathogen invasion in intestinal epithelial cells (IECs). Most TLRs are localized in the basolateral membrane of enterocytes, although TLR2 and 9 are also expressed on the luminal surface (Figure 2A) (82–84). TLRs mediate enterocyte-driven reactions against viral and bacterial attacks. On the contrary, tuft cells which are rare, are specialized cells that act as sensors for parasites. When an infection occurs, tuft cells induce ILC2 activation and expansion by production of IL25. In turn, ILC2 secrete IL13 causing tuft cell proliferation, amplifying the signal (Figure 2C). The tuft cell-ILC2 cross-talk determines a type 2 cytokine response, activating goblet cells, macrophages, eosinophils, and other effectors (85, 86).

The cytokines that have a primary role in gut homeostasis and damage are summarized in Table 2. These cytokines, produced by NK cells and ILC, are involved in the regulation of these cell types, of their function and in integrity of gut mucosa. IEC behavior is modulated by several inflammatory cytokines that can increase tight junction permeability (TNFα, IFNγ, IL1β, IL6) (86), priming the immune system and triggering a chronic response against innocent targets. To counteract inflammation, IECs chronically release soluble IL1β receptor (sIL1RII) and thymic stromal lymphopoietin (TSLP), while inflammatory cytokines trigger TGFβ neosynthesis. Soluble IL1RII neutralizes IL1β, while TSLP and TGFβ condition dendritic cells, promoting Th2 and regulatory T cell (Treg) differentiation. Interestingly, sIL1RII is strongly downregulated during the active phases of Crohn's disease (CD) (87–89). A third regulatory mechanism is represented by the autocrine IL10 loop, activated in IECs in response to IFNγ, which re-establishes the mucosal barrier and maintains immunotolerance and acts through IL10R (90–92). Indeed, IL10 KO mice develop a gut permeability defect that is associated with increased levels of TNFα and IFNγ, and exacerbates into a chronic colitis mediated by commensal bacteria (90–92).

**Table 2:** Cytokines involved in gut homeostasis and diseases.

**Cytokine**	**Producer cells**	**Target cells**	**Physiology**	**Disease**
IFNγ	NK, TL, ILC	Mϕ, BL, TL, IECs, END	Class II antigen expression, immunity, epithelial/endothelial permeabilization	CD, UC
IL1β	Mϕ, NK, PMN	Mϕ, BL, TL, IECs, END, FBs	Pyrogen, induces multiple inflammatory cytokines expression	Autoinflammatory disorders, CRC
IL6	Mϕ, IECs, END, FBs	BL, TL, FBs, HEP	Pyrogen, induces acute phase proteins, induces B and T cells maturation and platelet production	Chronic inflammation and autoimmunity, CRC
IL10	Mϕ, many tissues	most cells	Inhibits inflammatory and Th1 responses, stimulates BL, restores epithelial homeostasis	IBD, CRC
IL12	DCs, Mϕ, PMN	NK, TL, ILC, Tγδ	Th1 responses, TNFα and IFNγ secretion	IBD
IL13	NK, TL, ILC, EO	BL, Mϕ, IECs, FBs	Th2 responses against helminths, goblet cells activation and proliferation	CD, UC, fibrosis
IL15	Mϕ, DCs, IECs	NK, TL, ILC, Tγδ, IECs	Th1 responses, IEC death	CD, UD, coeliac disease
IL17	TL, NK, Tγδ, ILC	TL, IECs, END, FBs	Th17 responses, IEC barrier strength	CD
IL18	IECs	TL, NK, IECs	Limits Th17 differentiation, induces IFNγ secretion, sustains IEC regeneration, blocks goblet cells development	UC
IL22	ILC, NK, Tγδ, TL	IECs	Secretion of antibacterial peptides and mucins, LGR5^+^ stem cells proliferation	CD, CRC
IL23	DCs	ILC, NK, Tγδ, TL	IL22 and IL17 expression, Th17 responses	CD, CRC
IL25	Tuft cells, TL, Mast cells	ILC2	Th2 responses against helminths	
TGFβ	DCs, IECs, FBs	LT, IECs, FBs	Inhibits Th1 and Th2 responses, induces Th17 and Tregs, IgA maturation, FB proliferation, IEC inhibition	CD, UC, CRC
TNFα	Mϕ, TL, NK, PMN, EO, IEC, END, FBs	Mϕ, TL, PMN, IEC, END, FBs	Pyrogen, induces acute phase proteins, pro-inflammatory, cytotoxic responses, epithelial permeability and IEC apoptosis	CD, autoimmunity

*Mϕ, macrophages; NK, natural killer; TL, T lymphocytes; BL, B lymphocytes; ILC, innate lymphoid cells; Tγδ, gamma-delta T cells; DCs, dendritic cells; PMN, polymorphonucleated neutrophils; EO, eosinophils; IEC, intestinal epithelial cells; END, endothelial cells; FBs, fibroblasts; HEP, hepatocytes; CD, Crohn's Disease; UC, Ulcerative Colitis; CRC, Colorectal carcinoma; IBD, Inflammatory Bowel disease*.

In the gut, the specific subunit IL22R1 is almost exclusively expressed by IECs, making them a relevant target of IL22 produced by ILC, NK cells, CD4^+^, CD8^+^, and γδT lymphocytes. IL23, IL6, and IL1β are the main inducers of IL22, while TGFβ suppresses IL22 expression in all T cell subsets and induces IL17 expression. The main intestinal IL22-producing cells are NKp46 and RORγt positive (mouse), or NKp44 and RORC positive (human) (93). These do not express IL17 and lack typical NK cell effector functions (94). IL22 promotes the secretion of mucus-associated molecules, like MUC-1,−3, and−13 and other anti-microbial proteins, from goblet cells, reducing bacterial translocation across the epithelial barrier (95–100).

IL22 and IL17 frequently act in concert to limit bacterial invasion. The most common forms of IL17 are IL17A and IL17F, which share the same receptor but do not have completely overlapping activities. NKp46^+^RORγt^+^ NK and Tγδ innate immune cells are able to secrete IL17 and play an essential role before activation of a full Th17 response. The main source of IL17 is Th17 lymphocytes, which have differentiated from naïve CD4^+^ T cells by APCs secreting TGFβ, IL6, and IL21 and are activated by IL23. The IL17 receptor is expressed by leucocytes, IECs, vascular endothelial cells and FBs, and increases G-CSF, IL6, and IL8 release favoring granulopoiesis and neutrophil recruitment (Table 2 and Figure 2B). IL17 also contributes to strengthen the epithelial barrier by inducing tight junction formation in IECs. While an excess of IL17 contributes to CD, its presence is required to inhibit invasive bacteria (101, 102).

## Human NK Cells and Immune Response to Gut Infections

Most of what is known about the role of NK cells in response to gut infections comes from murine models (103–108). Murine NK cells appear to be relevant for *Listeria monocytogenes, Salmonella, Citrobacter rodentium*, and *Yersinia enterocolitica* infections (103–108). An efficient response to these infections mediated by NK cells is dependent on cytokines, such as IL15 and IFNγ. All molecular mechanisms involved in rodent gut immunity are very well reviewed elsewhere (108) and a specific analysis is beyond the scope of this review. It is conceivable that human NK cells in the gut can play a role in eliciting inflammation during bacterial infections that is independent of viral clearance and tumor control. Indeed, NK cells, like other innate cells, such as macrophages and neutrophils, can use different TLRs, mainly TLR2, TLR3, TLR4, and TLR9, to interact with bacteria-associated peptidoglycans, lipopolysaccharides, virus-derived dsRNA, and DNA with CpG motifs (also known as pathogen-associated molecular patterns, PAMPs) (109) to elicit an inflammatory response (Figure 2A). IL12 and IL18 produced by mucosa-associated macrophages are responsible for amplifying the immune response mediated by NK cells. In turn, IFNγ released by NK cells can trigger activation of myeloid cells to augment phagocytosis, respiratory burst and killing of bacteria (Figure 2B). These effects can further amplify the activation of NK cells and IFNγ toxicity, leading to systemic inflammation. Patients suffering from sepsis show dysfunction of several leukocyte subsets, including NK cells, that can cause a decreased host response against the primary bacteria and favor superinfections by other bacteria or latent viral reactivation, ultimately leading to fatal outcomes (110).

Focusing on NK cells in humans and NK cell-mediated anti-viral activity, interesting results analyzing the alteration of the mucosal distribution of NK cells during human immunodeficiency virus (HIV) infection have been reported (44). The frequency of NK cells is increased in HIV subjects with incomplete CD4^+^ T cell recovery in PB upon long-term anti-retroviral therapy. More importantly, spontaneous HIV controllers with protective KIR/HLA genotypes (111, 112) showed higher numbers of IEL NK cells than those in subjects with non-protective genotypes. This suggests that a peculiar NK cell subset displaying CD57 may be involved in control of HIV replication at the rectosigmoid mucosal site. Also, Human Herpesvirus 6 (HHV-6) can influence the NK cell-mediated response in the large bowel (113, 114). Indeed, HHV-6 shows a wide cell tropism *in vivo* and, as similar to other herpesviruses, causes a lifelong latent infection in humans and can be found in the large bowel. Notably, the HHV-6 products U51A and U83A suppress the surface expression of NKG2D and NKp30, two relevant activating receptors of NK cells (114). This suppression can impair the ability of NK cells to counteract HHV-6 reactivation and to recruit adaptive immune cells for elimination of the virus. In addition, some early viral proteins downregulate NKG2D ligands and transcription of B7-H6 mRNA, the reported cellular ligand of the activating receptor NKp30 (115) (Table 1). These findings suggest that the inefficient NK cell response can eventually lead to impaired clearance of HHV-6 and determine the establishment of a persistent infection (116).

## Gut microbiota and Immune Response: Diet and Probiotics

Disorders in the development or composition of bacterial microbiota (known as dysbiosis) result in immunological dysregulation, leading to altered immune responses that may underlie disorders such as IBD, allergies, and cancer. In turn, the term “probiotic” is used to describe dietary microbes that confer a health benefit to the host (117). In animal models, diet-modified microbiomes can rapidly promote obesity or reduce incidence of diabetes, in association with decreased pro-inflammatory cytokines IL17 and IL23 in colon mucosa (118).

Diet, gut microbiota, and immune responses are probable explanations for the expanding incidence of inflammatory/immune diseases such as asthma, type 1 diabetes, and IBD in people living in developed countries (119). A low fiber intake adversely affects the intestinal microbiota and leads to decreased production of immunomodulatory products, in particular the short-chain fatty acids (SCFAs) acetate, propionate, and butyrate, all of which are critically important for mucosal immune homeostasis and intestinal epithelial integrity (120). For example, butyrate has an anti-inflammatory effect by inhibiting the recruitment and pro-inflammatory activity of neutrophils, macrophages, and effector T cells, and increasing the number of Tregs. IBD patients have reduced SCFA-producing bacteria and reduced butyrate concentration linked to a marked increase in the number of pro-inflammatory immune cells in the gut mucosa (121).

Intestinal APCs protect the body against infections, and having co-evolved with microbiota, maintain immune tolerance to the normal gut microbiota. For example, DCs of Peyer's patches in mice produce high levels of IL10, and gut macrophages, located in close proximity to the intestinal microbiota, develop a non-inflammatory phenotype termed “inflammation anergy” when encountering microbial stimuli in homeostatic conditions (122).

Probiotic bacteria are considered to be “generally recognized as safe (GRAS)” organisms by the Food and Drug Administration in the United States (123). It is supposed that probiotic bacterial cells and/or their soluble factors exert immunomodulatory effects by activating TLR on gut DCs and macrophages, driving APCs to produce cytokines required for antigen-specific Th1 polarization, such as IL12, or leading to immunological tolerance. *Lactobacillus rhamnosus* GG (LGG) can relieve intestinal inflammation in patients with atopic dermatitis and food allergy by decreasing TNFα production and promoting IL10 expression (123, 124). In addition, LGG has also been shown to induce intestinal secretion of IgA in atopic children (124). A synbiotic (synergistic combinations of probiotic and prebiotic) comprising *Bifidobacterium longum* has been demonstrated to reduce CD in a randomized double-blind placebo-controlled study with an evident decrease in TNFα expression (125).

Along this line, *Lactobacillus plantarum* (*Lp*) can efficiently increase the expression of the natural cytotoxicity receptor (NCR) family and of IL22 in NK cells (126). Transfer of PB NK cells stimulated by *Lp* conferred protection against intestinal epithelial barrier damage induced by enterotoxigenic *Escherichia coli* (ETEC) in NCM460 cells *in vitro*. PB NK cells stimulated by *Lp* could partially offset the reduction in transepithelial electrical resistance (TEER) of NCM460 cell monolayers caused by ETEC. Furthermore, *Lp-*stimulated, compared to *Lp*-unstimulated, NK cells added to ETEC-infected NCM460 cells increased the expression of IL22R1, p-Stat3, and p-Tyk2 by NCM460 cells, which, together with ZO-1, claudin-1 and occludin, are known to play important roles in intestinal epithelial barrier function (127). Mechanistic experiments using polyclonal blocking anti-IL22 antibody showed that *Lp*-stimulated NK cells lost the ability to maintain TEER in NCM460 cells challenged with ETEC (127). These results suggest that *Lp* stimulation of NK cells could enhance IL22 production, which in turn provides defense against ETEC-induced damage to the intestinal epithelial barrier. Further studies demonstrated that treatment with different strains of *Lp* induces TRAIL on the cell surface of PBMCs. TRAIL production depended on IFNα and IFNγ and facilitated NK cell activity exerted by PBMCs against cancer cells (128).

Aging leads to a decline in immune function that adversely affects gut microbiota. In a prospective double-blind, randomized crossover study in 40 healthy elderly subjects (aged 60–80 years) consumption of *Lactobacillus rhamnosus* GG combined with soluble corn fiber increased NK cell activity, decreased the pro-inflammatory cytokine IL6 and decreased total and LDL cholesterol (129). In another phase II randomized, double-blinded, placebo controlled clinical trial, *Lactobacillus salivarius* treatment significantly increased the percentage of NK cells and monocytes, as well as the plasmatic levels of immunoglobulins and the regulatory cytokine IL10 (130).

The GI tract contains the largest endocrine organ in the body. Due to the strategic location of enteric endocrine cells in gut mucosa, interactions with the immune systems are very likely to play an important role in immune modulation (131). At least 14 different populations of enteric endocrine cells release biologically active compounds (132, 133). Although some data on the influence of these substances on Th1/Th2 responses in the gut are available (132, 133), to our knowledge information on NK cell recruitment and function is lacking.

## Inflammatory bowel disease (IBD) and NK cells

Overpowering cytokine production and chronic inflammation are typical of IBD, mainly represented by CD and ulcerative colitis (UC) (2, 134, 135). While the cause of IBD is still unknown, it is conceivable that associations between the altered immune response against intestinal flora and the genetic background of susceptible individuals could be responsible. Many studies have reported the linkage between the IBD3 region in the human leukocyte antigen (HLA) complex and CD or UC (136). Likewise, major histocompatibility complex (MHC) class I chain-related molecules (MIC) alleles and MICA polymorphisms have been associated with IBD [141, 142].

**Figure 3:**
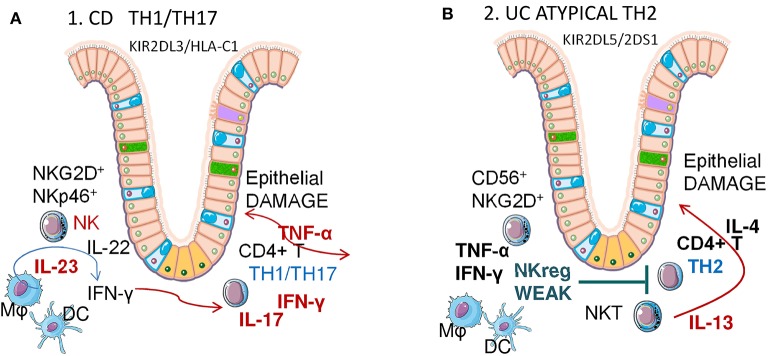
Crohn Disease (CD) and Ulcerative colitis (UC) pathogenesis: possible role of NK cells. Different Th1/Th2 responses in CD and UC. Mϕ, macrophages; DC, dendritic cells; NK, natural killer cells; NKT, natural killer-like T cells. **(A)** Crohn's Disease is mainly a Th1/Th17 IBD, linked to a KIR2DL3/HLA-C1 genotype in NK cells. Mucosal NK cells are RORC, CD127 (IL7Rα), NKG2D and NKp46 positive, produce IL22 and IFNγ when activated via IL23 and contribute to the expansion of CD4^+^ Th1/Th17 lymphocytes that, in turn, secrete both IFNγ and TNFα, in addition to IL17. **(B)** Ulcerative Colitis is mainly an atypical Th2 IBD, linked to a KIR2DL5/2DS1 genotype in NK cells. Atypical NKT produce IL13, which induces epithelial damage. In turn, NK cells are unable to induce an efficient TNFα IFNγ response to reduce the Th1/Th2 imbalance, contrasting excessive IL13 secretion.

There is evidence that supports a major role for adaptive immunity in the pathogenesis of IBD, involving Th1 and Th2 cells, together with other subsets of T cells, namely Th17 and Tregs (134, 135). In particular, while it has long been thought that CD is due to an abnormal Th1 response with increased secretion of the pro-inflammatory cytokines IFNγ TNFα and IL1β (Figure 3A), UC has been associated with a non-conventional Th2 response that involves IL5, IL6, and IL13 (Figure 3B) (134, 135). Besides classical Th1 and Th2 responses, Th17 cells, which are a subset of T lymphocytes that expand in the presence of IL23, can also contribute to IBD pathogenesis through IL17 production (137). More recently it has been shown that the innate arm of immune response is relevant to the favoring of gut inflammation in IBD patients (137). While altered epithelial barrier function has been described in patients with CD and UC, it remains unclear whether these lesions represent the cause or the effect of chronic inflammation with intensified production of cytokines. Among the highly produced cytokines, IL23, besides amplifying the IL17 circuit, can also act on innate immune cells or unconventional T lymphocytes, such as γδT lymphocytes which are good producers of IL17, or NK cells (138). In this regard, the presence of killer (K) cells in mesenteric LN was described in CD patients long ago (139). The involvement of NK cells in IBD pathogenesis has been supported by a recent study on polymorphisms of killer immunoglobulin-like receptors (KIR) genes (140). KIR are NK cell surface receptors, which bind to the class I MHC and have inhibitory or activating effects on NK cells (Table 1). A meta-analysis of 432 UC and 1677 CD patients showed positive associations between 2DL5/2DS1 (members of KIR genes) and UC risk, and a negative association between 2DS3 and CD risk (140).

### Crohn Disease (CD)

Aberrant innate immune responses, such as huge antimicrobial peptide production and enhanced innate microbial sensing and autophagy, are associated with CD pathogenesis (134). How NK cells contribute to this uncontrolled immune amplification is still unclear, nevertheless several groups have recently identified a unique subset of mucosal NK cells that contributes to local immunity. These mucosal NK cells in the human gut are distinct from conventional NK cells and are characterized by expression of the transcription factor retinoic acid-related orphan receptor C (RORC), CD127 (IL7Rα), and NKp44 or NKp46 (Table 1). Moreover, NKp44^+^ NK cells produce IL22 (141), however whether they participate in pathologic or protective processes of chronic inflammation *in vivo* remains controversial. In humans, CD56^+^CD127^+^ NK cells are generated from LTi cells and produce little IFNγ, whereas CD56^+^CD127^−^ NK cells produce a large amount of this cytokine. NKp44 and NKp46 are expressed differentially on NK cells in the CD intestine, NKp46^+^ NK cells predominate in intestinal mucosa of patients with CD compared with patients with UC and with controls. Upon interaction with intestinal inflammatory macrophages, NKp46^+^NK cells from patients with CD are activated via IL23 and produce IFNγ (93).

Of note, genetic alterations in regulatory NK cell receptors have been reported. Indeed, KIR polymorphism is implicated in susceptibility to CD (140), with a significant association of the KIR2DL3/HLA-C1 genotype and CD (142), although the cellular mechanism of this genetic contribution is poorly defined. It has been described that the “licensing” of NK cells, determined by the presence of KIR2DL3 and homozygous HLA-C1 in the host genome, results in cytokine reprogramming that permits promotion of CD4^+^ T cell activation and Th17 differentiation *ex vivo*. Licensed NK cells are more polarized to pro-inflammatory cytokine production than unlicensed NK cells. These cytokines, including IFNγ, TNFα, and IL6 (Table 2), augment CD4^+^ T cell proliferation and IL17A/IL22 production. Interestingly, antibody blocking of these cytokines could reduce their effect (143), presenting a potential therapeutic target for CD and other IBD (144). However, due to the complexity of the cytokine network involved and the fact that Th17 cells may also have protective functions, neutralization of IL17A failed to induce any improvement in CD, at variance with other autoimmune disorders (145, 146).

An exploratory clinical trial to investigate the safety and efficacy of the humanized anti-IL6R mAb tocilizumab (also known as MRA) in patients with CD, showed promising results, with 20% of the patients entering remission and acute-phase responses normalized by a single MRA infusion (147). However, the gold standard IBD treatment, including CD, for many years has been based on the use of humanized or human anti-TNFα antibodies, despite many adverse effects—including the risk of tuberculosis (148).

Since CD pathogenesis has been linked with the IL12/23 pathway (138, 143, 149), a recent novel approach to interrupt this pathway has been proposed using ustekinumab, a therapeutic monoclonal antibody that blocks the p40 subunit of both IL12 and IL23 and prevents interactions with their receptors on T, NK, and APCs, has established efficacy in psoriasis (150).

Another important mechanism in the pathogenesis of CD is the expression of stress-related molecules belonging to the MIC family (136, 151). These molecules are recognized by NKG2D receptors (Table 1) expressed on T and NK cells and induce activation of these cytolytic cell types, thus contributing to mucosal cell damage (152, 153). A recent phase II clinical trial showed that an antibody against NKG2D induced clinical remission of CD in some patients, suggesting NKG2D and its ligands are attractive new targets for IBD therapies (152, 154).

### Ulcerative Colitis (UC)

UC is characterized by contiguous inflammation of colonic LP wherein damage is triggered by an over-response to bacterial antigens, enhancement of DC and macrophage stimulation via TLRs (155, 156). In UC, the T-cell response to antigens is not Th1 dominant, as in the case of CD, but rather is either Th2 (IL4, IL13) dominant, or is mediated by specialized cells such as natural killer-like T (NKT) cells producing IL13 (Figure 3B). LP-NKT cells from UC patients produce significantly greater amounts of IL13. Thus, UC pathogenesis is considered to be an atypical Th2 response mediated by a distinct subset of NKT cells that produce IL13 and damage epithelial cells (156, 157). Along this line, decreasing IL13 production following treatment with IFNβ1a is associated with clinical improvement of UC symptoms (158). Other inflammatory cytokines (TNFα, IL1, IL6, IL9) play significant roles in worsening, while anti-inflammatory cytokines (TGFβ, IL10) delay disease progression (156).

In this context, the actual pathogenetic role of NK cell is still undefined. KIR polymorphism and positive associations between 2DL5/2DS1 KIR and UC risk has been reported (140, 159). In active UC, peripheral NK cells were decreased significantly compared to inactive UC. After anti-TNF treatment, peripheral NK cells in responsive IBD patients were significantly higher than in non-responsive UC (160). Intestinal LP NKG2D^+^ NK cells have been investigated in UC, since it is thought that they play a role in regulating Th1/Th2 balance (Figure 3B). Severe UC patients have higher expression of mucosal NKG2D and its ligand MICA, and a lower number of LP NKG2D^+^NK cells than mild to moderate UC. Furthermore, in bioinformatics analyses, mucosal Th1 cytokines, mainly TNFα, emerged as crucial to CD, but not UC, since anti-TNFα treatment proved less effective than in CD. This would suggest that NKG2D^+^ NK cells play a regulatory role in UC by secreting Th1 cytokines that modulate the Th2-predominant Th1/Th2 imbalance (160). Although the precise role of NKG2D is less clear in UC pathogenesis than in CD, this molecule should be considered as a possible therapeutic target as well (153). Finally, NK cells can be targeted in CD by 6-mercaptopurine, a drug also used in UC treatment that causes NK cell apoptosis and depletion, which possibly limits the inflammatory response (161).

## NK Cells in Colorectal Cancer (CRC)

CRC has been recently subdivided into four consensus molecular subtypes (CMS) on the basis of genetic and microenvironmental signatures. Three of these classes recall the well-known definitions of microsatellite instable (CMS1), sporadic (CMS2) and stromal rich (CMS4) CRC, while the class of metabolic CRC (CMS3) was recently introduced. In CMS2 and 3, comprising about 50% of all CRC, immune and stromal infiltration are limited. CMS1 (14%), due to microsatellite instability (MSI), has a high DNA mutation rate, causing the production of altered antigens that trigger the immune response. Accordingly, CMS1 shows a strong infiltration of innate and adaptive immune cells. It is not by chance that only MSI-high CRC showed a clinical response to anti-PD-1 and anti-PD-L1 immune checkpoint inhibitors therapy (162, 163). CMS4 (23%) defines a particular group of CRC showing a strong infiltration of stromal cells, such as FBs, an intermediate infiltration of immune cells and a minority of tumor cells. Despite the low prevalence of tumor cells, this CRC subtype has the worst prognosis. CMS4 is characterized by high TGFβ expression, determining an immunosuppressive microenvironment that is enriched in regulatory cells (164). In this regard, CRC-associated FBs co-cultured *in vitro* with NK cells can downregulate the expression of NKG2D and the NKG2D-mediated recognition of tumor target cells by NK cells (165).

**Figure 4:**
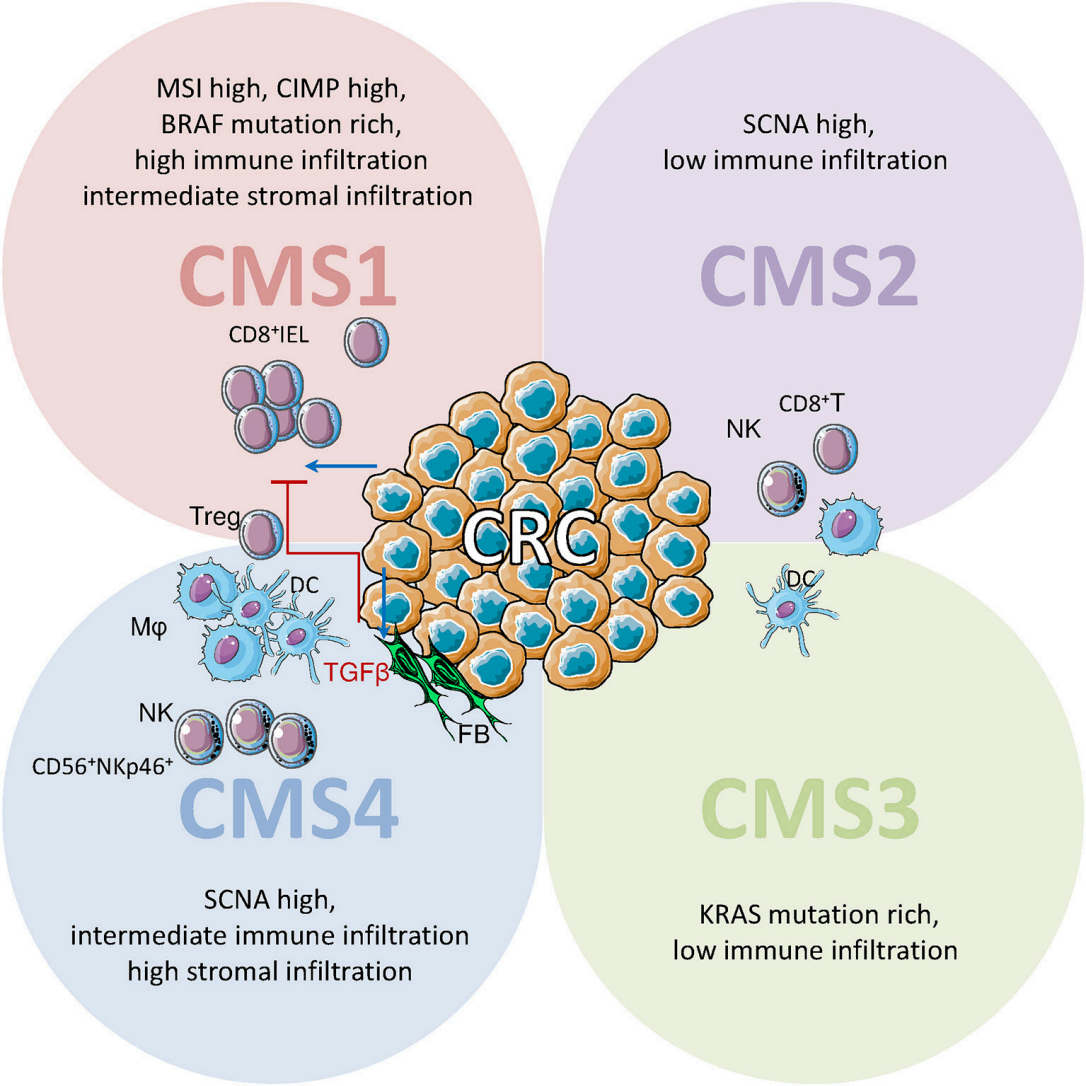
Immune cell infiltrate in CRC: possible role of NK cells. Schematic representation of immune cell infiltrate in CRC defined on the basis of gene expression profiles in consensus molecular subtypes (CMS). IELs: intraepithelial lymphocytes; Mϕ, macrophages; DCs, dendritic cells; NK, natural killer cells; FBs, fibroblasts. IELs are CD8^+^ cytolytic T lymphocytes (CTL), while CD56^+^NKp46^+^ NK cells are scattered in the stroma. CMS1 (14% of CRC, upper left) are characterized by high somatic copy number alteration (SCNA), high CpG island methylator phenotype CIMP), and a strong microsatellite instability (MSI); this high DNA mutation rate leads to altered antigens that trigger immune responses and causes a strong infiltration of innate and adaptive immune cells. In CMS2 and CMS3 (50% of CRC, upper right and lower right) immune and stromal infiltration are limited. CMS4 (23% of CRC, lower left) shows a strong infiltration of stromal cells (FBs) and intermediate infiltration of immune cells. In CMS4 high TGFβ expression determines an immunosuppressive microenvironment enriched in regulatory T cells.

CRC frequently shows diminished MHC class I expression, that would increase tumor susceptibility to attack by NK cells. In CRC with partial or total HLA class I loss, effector IELs are CD8^+^ CTL, while NK (CD56^+^) cells are only observed scattered in the stroma. On the contrary, in normal mucosa, NKp46^+^ CD3^−^ NK cells can also show intraepithelial localization and typically coexpress CD57 (46, 166) (Figure 4). To our knowledge, immunohistochemical studies on the NK cell population of CRC systematically used anti-NKp46 antibodies, thus no information about the NKp44^+^ population is available. The quantification of NKp44^+^ cells in the tumor infiltrate of CRC patients could be of great interest, as it has been shown that CRC-initiating cells preferentially express ligands for NKp30 and NKp44 (93, 167). CRC shows a reduced number of infiltrating NK (NKp46^+^) cells compared to normal mucosa, also when CD8^+^ T cell numbers are elevated, suggesting that CRC is able to limit NK cell infiltration. This limitation of NK cell recruitment is not mediated by the absence of homing chemokines as CXCL9, CXCL10, CCL3, CCL4 (active on CD56^bright^ NK cells) and CXCL8, CXCL1, CXCL5, CXCL12 (active on CD56^dull^ NK cells) show higher concentrations in the tumor than in the unaffected mucosa. Despite the reduced number in CRC, the NKp46^+^CD3^−^ NK population is enriched in CD16^+^CD56^+^ cells compared to normal mucosa, suggesting that ADCC could be elicited by humanized Ab targeting tumor cells (76, 165). The contemporary infiltration of CD8^+^ T and NK cells in CRC is apparently linked to a better prognosis compared to the infiltration of CD8^+^ T cells only. This effect could be due to direct involvement of NK effectors, or represent a less immunosuppressive microenvironment of the tumor (168). A proof of principle for the efficacy of NK cell reactions against CRC comes from a recent case-report describing a complete and sustained response mediated by NK cell activation, in a metastatic CRC patient (169). Indeed PVR (CD155), Nectin-2 (CD112), and MICA/B, the ligands for the activating NK receptors DNAM1 and NKG2D, are expressed in CRC, suggesting a possible target for NK effectors. Though CRC infiltrating NK cells show a partially reduced expression of DNAM1 and NKG2D, increased soluble (s)CD155, and sMICA/B have been detected in patient serum (170).

A TMA study on 462 primary colorectal tumors evaluated MIC, ULBP, RAET (NKG2D ligands) and the NK cell infiltration. The higher expression of all ligands was found in stage I (UICC-TNM) tumors, becoming less frequent in advanced stages. MIC levels correlated to NK infiltration. The contemporaneous high expression of MIC and RAET1G was linked to improved patient survival of 77 months over CRC expressing one ligand or low levels of both (171). According to these observations, the future clinical application of NK-based immunotherapies against CRC apparently depends on the identification and neutralization of the tumor-derived mediators that limit NK cell infiltration. As these mediators do not apparently influence CD8^+^ T lymphocyte recruitment, they should probably be looked for among signaling molecules specific for NK cells. Among them, KIR and CD16 have been implicated in defining CRC genetic risk and clinical stage, although the matter is still under debate (172–174). Indeed, the allele frequency of KIR2DL2 and KIR2DS2, in the absence of their cognate HLA-C1 ligands, were significantly associated with reduced genetic risk of CRC. Conversely, CD16-48H polymorphism was associated with increased genetic risk of CRC (172). In 1990 Adachi et al. reported a higher number of CD57^+^ NK cells in draining CRC LN than in primary or metastatic lesions and suggested that these cells can limit tumor spreading (175). Indeed, acquisition of CD57 represents a shift toward a higher cytotoxic capacity, greater responsiveness to signaling via CD16 and NCR and decreased responsiveness to cytokines. This would be consistent with enhanced tumor surveillance/cytotoxicity of the mature, CD57^+^ NK cell subset (46).

As discussed above, mucosal epithelium is a source and a target of several cytokines (Table 2) (176). In particular, IL18 is a typical cytokine produced by the normal mucosa and is decreased in CRC: its downregulation frequently correlates with a lack of IFNγ and FAS ligand, and formation of metastases. IL18 primes NK cells *in vivo* to produce IFNγ upon stimulation with IL12 and increases IFNγ neosynthesis in NK cells activated through CD16. In mouse models, IL18 limits the differentiation of Th17 cells and sustains epithelial regeneration upon inflammatory damage (177–179). IL22, produced by ILC, NK and T lymphocytes, binds only to IECs, representing a more defined target for CRC therapies. Indeed, IL22 has been associated with CRC growth, tumor cells protection from cytotoxic and apoptotic effects of chemotherapy and FOLFOX resistance in CRC patients (180–182).

IL12 is formed by IL12p35 and IL12p40 subunits. IL12p40 can also form a homodimer, antagonizing IL12 activity, or bind IL23p19 to form IL23; hence the overall effect of the single cytokine can be elusive. NK and γδ T cells express high levels of IL12 receptor. IL12 plays a central role in Th1 responses, triggering the activation of NK and CD8^+^ T cells and inducing IFNγ production. In CRC patients, a low production of IL12 in DCs was associated with a poor prognosis (183–185). Despite the shared subunit with IL12p40, IL23 activates local inflammatory responses not involving Th1 effectors. IL23 is secreted by DCs, macrophages, and neutrophils during gut inflammation (Table 2). The main intestinal targets of IL23 are Th17, ILCs, and Tregs, inducing the production of IL22 and IL17. High IL23 levels, coupled with low SOCS3 expression in primary CRC, were predictive of increased risk of metastasis (186).

Despite the lack of data on NK cells and IL17, this cytokine is of interest as it shows a controversial influence on CRC. A genetic clustering of 125 CRC showed reduced survival of patients with a high Th17 signature. However, a tissue microarray evaluation of IL17^+^ cells in 1148 CRC samples did not confirm this observation, as IL17 positive staining was correlated with neutrophils and CD8^+^ cytotoxic lymphocytes infiltration, and intraepithelial Th17 lymphocytes were linked to a favorable prognosis. Thus, the overall effect of IL17 is apparently linked to the localization of IL17-producing cells. A further complication in the definition of the pro-tumor properties of IL17 comes from the IL17A polymorphism rs2275913 (G197A), with the AG and AA genotypes strongly associated to an increased CRC incidence (187–191).

## NK/Immune Cell-Exosomes Connections: Role of Signals Originating in Gut Cells or Microbiota

The importance of extracellular nanovescicles (EVs), ranging from 50 to 1,000 nm in size, exosomes in particular (size 100–200 nm), as biological vehicles able to affect distant organs is well established. For example, salivary exosomes from patients with IBD, a condition predisposing toward UC and CRC, carry larger amounts of proteasome subunit alpha type 7 (PSMA7) than those in healthy subjects. High-throughput sequencing revealed ~850 proteins in EVs secreted by intestinal cells and present in ascites of patients with CRC, showing that intestinal EVs may be transferred between organs and, in turn, modify the composition of EVs released by the target tissue (192). For a thorough examination of the state of the art of characterization and use of exosomal vesicles, we refer to recent comprehensive reviews (193, 194).

NK and organ cells apparently communicate in forward and backwards signaling loops where cell- and NK-derived exosomes exert reciprocal control in the circuit. The impact of intestinal physiology, depending also on the balance of the gut microbiota, on the immune system at the whole-body level has recently emerged as a crucial topic in cancer research and in research of related conditions (chronic inflammation, angiogenesis and metabolic syndrome, to cite a few). Responses to immunotherapy and metastatic dissemination seem to depend, at least in part, on the gut microflora-host interplay. In a remarkable *in vivo* preclinical study, the exodus of gut-primed immune cells in mice engineered with the fluorescent Kaede protein provided evidence of an intense two-way trafficking from defined colon tracts to lymphoid organs. Also specific LN at sites distant from the intestine, besides mesenteric LN, were the destination of Tregs, Th17, and other innate and adaptive immune cells involved in chronic intestinal and systemic inflammation (195). This work highlights the powerful potential of gut homeostasis and dysbiosis on control of health; however, a possible role for gut or immune cell EVs in these conditioning pathways was not examined. The influence of gut exosomes on the immune system is currently being intensely investigated, with efforts primarily focused on intestinal DC EVs and adaptive immunity cross-talk (196). Several experimental models have advanced knowledge of the effects of intestinal mucosa EVs on innate immune cells (mostly neutrophils, monocytes and macrophages), and vice versa, in inflammatory diseases of the GI tract (197).

Indeed, different preclinical studies have shown that tumor EVs promote tumor cell organotropism, favoring a pre-metastatic niche in the host microenvironment. A role for tumor EVs in metastatic progression has been reported for CRC, gastric (GC), and pancreatic ductal adenocarcinoma (PDAC) invading the liver microenvironment. Stromal cells seem to be the privileged targets of tumor exosomes in the metastatic organ, establishing a pro-inflammatory and immunosuppressive state (198).

### Effects of Cellular Exosomes on NK Cell Function

The data reported focus primarily on NK cell activation induced by exosomes released by DCs, stressed cells, or tumor cells. EVs, particularly tumor EVs, are endowed with anti- or pro-tumor potential, and a dual immunostimulatory or immunosuppressive role. NK cell activation is only one of the multiple and contrasting functions of cellular EVs, and the final outcome at the level of the organ and the whole body, as well as on the disease state, comes from the prevailing effect.

DC-derived exosomes (Dexs) can boost NK effector functions through the binding of Dexs carrying TNFα to TNFR on NK cells, which stimulates the release of IFNγ and the cytotoxic response (199). Dexs have been shown to trigger NKG2D-dependent NK cell expansion and activation in lymphoid organs in mice (200). Moreover, Dexs express the nuclear/membrane protein BAG6, a ligand for the activating NCR NKp30 (Table 1). BAG6-expressing Dexs induce cytokine release by NK cells and their effector program (201). Dexs have the ability to directly kill T and NK cells, via Fas-L, TRAIL and other death receptors (199). As we will discuss later, Dexs can also kill tumor cells by similar mechanisms, opening new ways for anticancer therapies to target cancers.

A prominent immunosuppressive function for tumor EVs is apparently mediated by TGFβ1 and NKG2D ligands. It is well-established that intestinal epithelial cell EVs carrying abundant αvβ6 integrins can prime DCs and Tregs to produce active TGFβ from the latent form, thus acquiring a tolerogenic phenotype (202). Nevertheless, the stress-induced heat shock proteins (Hsps) are known to confer tumor immunogenicity and induce NK antitumor responses (203), and EVs expressing high levels of Hsp60 have been found in CRC. Exosomes carrying abundant Hsps have been shown to effectively limit liver metastasis in CRC and gastric cancer (204).

Pharmacological stress triggered by anticancer drugs, such as carboplatin and irinotecan, can also induce exosomal Hsps in hepatic carcinoma that can trigger NK antitumor activation. Membrane-bound Hsp70 is a tumor structure that enhances the cytotoxic attack by NK cells, improving their effectiveness. CRC EVs expressing Hsp70 can induce activating NK cell receptors, such as CD69, NKG2D, NKp44, and down-regulate the inhibitory NK receptor CD94, enhancing granzyme B-mediated NK cytotoxicity (204, 205). Tumor exosomes have been found to frequently exert immunostimulatory effects through the expression of BAG6/BAT3, a ligand for NK cell NKp30 activating receptor, normally expressed in DCs, as mentioned above.

The data available on the interactions between tissue or NK EVs in the gut are scarce. GI epithelial cells secrete EVs (206) and EV recipient cells include intestinal macrophages and DCs in the LP, which acquire information from the epithelial cells themselves or from luminal antigens provided by goblet cells. EVs secreted by intestinal mucosa, expressing intestinal epithelial-specific markers (A33, villin-1) and PGE_2_, can deliver their content to APCs or NKT cells in the liver. This initiates an immunosuppressive program in the organ and an anergic-like state in NKT cells that can inhibit the anti-tumor response. The immunosuppressive effects of intestinal EVs, however, could also be exploited to limit liver autoimmune attack (207).

### Effects of DCs and NK Exosomes on Neighboring Cells

Dexs, which have been thoroughly characterized, retain and expose proteins, ranging from presentation molecules (class I and class II MHC peptide complexes and CD1), costimulatory molecules (CD86, CD40), adhesion (ICAMs), and docking molecules (integrins), on the outer membrane. Clinical grade Dexs are of great value as more effective inducers of tumor-associated antigen (TAA)-specific T cell responses than DCs in novel immunotherapy strategies, and are currently being evaluated in clinical trials for vesicle-based cancer treatment (208).

Exosomes derived from NK cells were described in the context of immune surveillance against infectious agents and tumor cells by Lugini et al. (209, 210). Typical NK cells (CD56^+^CD16^+^CD3^−^) constitutively release exosomes in resting and activated states. NK vesicles can express the basal NK cell markers CD56, NKG2D, and, to a lesser extent, the NCR NKp46, NKp44 and NKp30, and the cytotoxic machinery of perforins and FASL to kill cancer cells. FASL, however, was undetectable in circulating serum exosomes (209).

Jong et al. recently showed that EVs released by activated NK cells induce caspase-3,−7 and−9, exerting a cytotoxic against a panel of different tumor cell lines (211). Besides perforin, these vesicles also contain granulysin, granzymes A and B. In this scale-up isolation procedure, activation and expansion of NK cells was obtained by incubation of PBMCs with artificial APCs, K562-mbIL21, expressing a membrane variant of IL21. NK cell expansion offers opportunities for preclinical studies and possibly for clinical applications in the future. Interestingly, an observational clinical trial at the Ottawa Hospital is presently recruiting healthy donors and CRC patients after surgery to measure NK cell activity by the classical Cr^51^ cytotoxicity assay. The scope of the study is to investigate the activation state of NK cells, in order to boost clearance of tumor metastasis by NK cells (212).

The potential theranostic uses of EVs, regardless of the cell from which they are secreted, are far from being fully exploited. The term “exosomes,” however, has been reported in 99 clinical studies, covering 47 observational studies aimed at defining disease-specific molecular signatures and intercellular signaling of exosomal vesicles. Further studies on exosomes are needed to develop their possible use as potential diagnostic markers or therapeutic tools for drug delivery. As an example, the exosomal form of KIR might be a valuable indicator of gut disease stage and/or progression.

## Future Perspective to Study NK Cells in Human Gut

The evidence outlined herein underscores the intrinsic difficulty in studying either the physiological or pathogenic role of NK cells in the human gut. Indeed, the complexity of the gut includes the cellular composition, the diet, and microbiota, the repertoire of hormones and cyto-chemokines, besides individual genetic background. On the other hand, NK cells and ILC are really rare and their precise identification is not easy. Nevertheless, the generation of organoids from colonic specimens and artificial scaffolds of the intestinal mucosa (213–215) suggest the possibility of studying the interactions of the NK-gut microenvironment in a more controlled experimental system. This approach might better define the relative contribution and relevance of NK cells and innate lymphoid cells in gut health and disease.

## Author Contributions

Although all the authors are responsible for and edited the whole content of this paper, some parts of the paper have been cured by a specific author. Briefly, AP wrote the introduction, NK cell features, mechanisms of localization of NK cells, some points on gut anatomy and CRC. RB, RV, and DC wrote the cytokines cross-talk, gut anatomy and CRC. NF wrote microbioma and endocrine influence on innate immunity, some mechanisms of antibacteria immune response. MRZ wrote the section on autoimmune diseases such as Chron Disease and Ulcerative Colitis. FT wrote the section on exosomes.

### Conflict of Interest Statement

The authors declare that the research was conducted in the absence of any commercial or financial relationships that could be construed as a potential conflict of interest.
